# Feasibility of Vigorous Extended Reality Tele-Exergaming for Cardiometabolic Health in Youth With Mobility Disabilities: Protocol for a Case Series Study

**DOI:** 10.2196/85246

**Published:** 2026-02-27

**Authors:** Byron Lai, Maggie Logan, Raven Young, Ashley Wright, Jordyn Terrell, Larsen Bright, Drew Davis, Christen J Mendonca

**Affiliations:** 1 Division of Pediatric Rehabilitation Medicine, Department of Pediatrics, University of Alabama at Birmingham, 1720 2nd Ave South, Birmingham, AL, 35294-1801, United States, 1 205-934-4011; 2 Department of Occupational Therapy, University of Alabama at Birmingham, Birmingham, AL, United States; 3 Department of Physical Therapy, University of Alabama at Birmingham, Birmingham, AL, United States; 4 Research Collaborative, School of Health Professions, University of Alabama at Birmingham, Birmingham, AL, United States

**Keywords:** active video gaming, exercise, disability, physical activity, exergaming, extended reality

## Abstract

**Background:**

Young people with mobility disabilities have limited options to maintain their cardiometabolic health and cardiorespiratory fitness. Active video gaming using extended reality head-mounted displays is becoming increasingly common for promoting serious exergaming. However, there is a need to identify dosing protocols that can potentially lead to meaningful improvements in cardiometabolic health and cardiorespiratory fitness.

**Objective:**

This feasibility study aims to explore potential benefits of vigorous-intensity extended reality exergaming, conducted at home with telemonitoring for body composition and cardiometabolic health, in 4 young people with cerebral palsy and overweight or obesity. The secondary aim is to assess the effects of the program on cardiorespiratory fitness. The tertiary aim is to describe the safety and acceptability of the intervention.

**Methods:**

This case study is a phase 1 feasibility trial with a pretest-to-posttest design including 4 participants. Young people with cerebral palsy and overweight or obesity (aged 13-24 years) will be purposively selected to participate based on 2 mobility categories (ambulatory: n=2; nonambulatory: n=2). The intervention prescription will include 240 minutes per week of vigorous-intensity exercise at home for a total of 6 weeks, with telemonitoring of exercise data that will be supplemented with weekly coaching calls. Participants will exercise using a head-mounted display to control an immersive exergame. Caregivers will agree to manage a play schedule and assess their children’s safety during play. Body composition will be measured using dual-energy x-ray absorptiometry at the pre- and postintervention time points (weeks 0 and 7, respectively). Blood-related health (fasting insulin, lipids, high-sensitivity C-reactive protein, and glycated hemoglobin) will be measured via a blood spot test, and blood pressure will be measured via a sphygmomanometer. Cardiorespiratory fitness (peak oxygen consumption) will be measured using a portable metabolic cart (COSMED K5) during a graded exercise test on an arm ergometer at the pre- and postintervention time points. Quantitative process metrics and qualitative feedback will be used to assess feasibility. Changes in outcomes over time will be descriptively analyzed.

**Results:**

Recruitment began in October 2025. All data are anticipated to be collected by December 2025. Full results are anticipated to be analyzed and submitted for publication by April 2026.

**Conclusions:**

This feasibility study tests an accessible and intensive program, which leverages high-intensity exercise gaming with telehealth procedures for children with cerebral palsy. Study findings will inform a pilot efficacy trial to improve cardiometabolic health and fitness among young people with mobility disabilities.

## Introduction

### Physical Activity, Disability, and Early Disease Risk

As children and adolescents with cerebral palsy (CP) transition into adulthood, their risk of cardiometabolic disease increases exponentially compared to their peers without disabilities [1-5]. This is thought to be a result of living a physically inactive lifestyle [6-9] owing to a lack of accessible exercise options and supportive environments [10,11]. While health professionals generally recommend more than 150 minutes of moderate-intensity physical activity per week for adults and more than 60 minutes of moderate to vigorous physical activity per day for children to improve and manage cardiometabolic health, most young people with physical disabilities are not meeting these guidelines [11-15].

### Limited Options for Exercise

For people with mobility disabilities, there is substantial evidence supporting the improvement of cardiorespiratory fitness, muscle strength and function, and gross motor skills through exercise [11]. However, there are limited studies demonstrating the positive effects of exercise on cardiometabolic health, particularly among children and adolescents with various disabilities [11]. To achieve improvements in cardiometabolic health, individuals need to engage in moderate to vigorous exercise regularly over a minimum of 1 to 3 months [16,17]. CP is characterized by disorders of movement and posture due to disturbances of a fetal or developing brain, which can include a variety of secondary complications such as epilepsy, pain, or musculoskeletal issues [18]. Therefore, individuals with CP typically encounter difficulty in long-duration participation in common modes of aerobic exercise, such as walking, running, and cycling.

### Extended Reality Exergaming

Exergaming is an exercise strategy that blends video games with serious exercise programming to improve health. Extended reality (XR) includes virtual, augmented, and mixed reality applications. For young people with various disabilities, XR has been used in exercise research to enhance motor and executive functions, physical fitness, movement quality, spatial orientation, and mobility, as well as to manage pain [19-24]. XR using a head-mounted display (HMD) is a common and commercially available method of exergaming that enables the user to engage with simulated environments by moving their body to play. HMDs can be used in both sitting and standing positions and have been found to elicit moderate- to vigorous-intensity exercise among children and youth with various disabilities [19,23,25].

### Telehealth and XR Exergaming

Telehealth programs include virtual communication and monitoring to provide a sense of accountability, which fosters strong adherence to home-based interventions [26]. Combining telehealth strategies with home-based exergaming has the potential to engage children and adolescents with mobility disabilities in high doses of exercise that are needed to improve cardiometabolic health. Additional advantages of using telehealth strategies with exergaming could include increased social support, cost-effectiveness, access to services, and reduced trainer burden [27]. Given that HMDs can be obtained at any major retailer, telehealth exergaming could address a critical limitation in exercise research and disability: confirmatory sample sizes to achieve generalizable study findings [11,23]. Although studies have examined the effects of play using XR HMDs to elicit moderate-intensity exercise [28], to the best of our knowledge, no published study has explored the effects of a high-volume and vigorous-intensity exercise program on objective biomarkers of cardiometabolic health among youth with CP. Therefore, this study has three purposes:

1. Describe the feasibility of high-volume, vigorous-intensity XR exergaming in 4 youth with CP who are overweight or obese through metrics related to participant safety and adherence to the exercise prescription
2. Explore the potential benefits of high-volume, vigorous-intensity XR exergaming for 6 weeks for cardiometabolic health and cardiorespiratory fitness in 4 youth with mobility disabilities who are overweight or obese
3. Describe the acceptability of the intervention through participants’ perceptions of completing the program via qualitative semistructured interviews

## Methods

### Study Design and Overview

This case study of feasibility uses a pretest-to-posttest trial design with a single group. The intervention will be conducted remotely for 6 weeks. Data collection will take place on-site at a university research laboratory in intervention week 0 and week 7. This study will include a separate (ie, not mixed methods) descriptive analysis of both quantitative and qualitative data.

### Participants and Recruitment

Recruitment will take place through word of mouth and patient referrals from the Division of Pediatric Rehabilitation Medicine at Children’s of Alabama. The study will purposively select a total of 4 youth with CP from the following 2 mobility groups: 2 who are ambulatory (Gross Motor Function Classification System [GMFCS] level I-II) and 2 who are nonambulatory (GMFCS level III-IV).

#### Inclusion Criteria

Inclusion criteria are as follows:

- Age of 13 to 25 years (to account for the World Health Organization definition of youth [15-25 years] and the previous minimum age of 13 years that was specified by the HMD manufacturer [now revised to 10 years])
- Overweight or obesity (based on Centers for Disease Control and Prevention BMI normative ranges for age and sex)
- Physical inactivity (<150 minutes per week of moderate exercise)
- GMFCS levels I to IV
- Wi-Fi internet access at home
- Physician clearance to participate

#### Exclusion Criteria

Exclusion criteria are as follows:

- Inability to communicate in English
- Complete blindness or deafness
- Propensity to photosensitive seizures
- GMFCS level V

### Equipment and Monitoring

**Figure 1.**
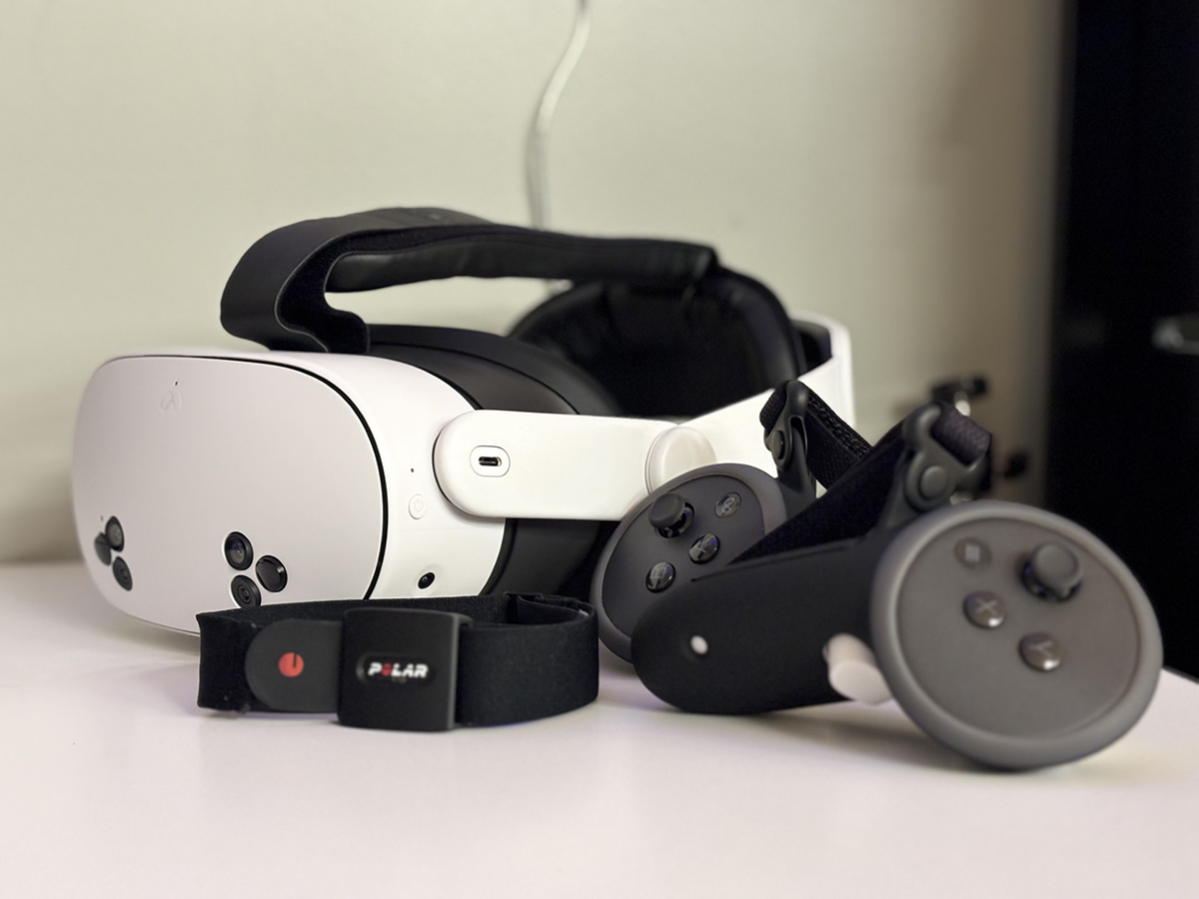
Meta Quest 3 head-mounted display with controllers and a Polar Sense heart rate monitor.

This study will use the Meta Quest 3S (Reality Labs) for active video gaming. This is a commercially available video gaming system. The system includes an HMD and 2 handheld controllers (Figure 1).

Heart rate data will be recorded and transmitted using a heart rate monitoring device (Polar Verity Sense) and mobile app (Polar Beat; Figure 1). The heart rate device will be placed on an armband and worn on the forearm. Participants will record their heart rate during exercise using the Polar Verity Sense, which will be uploaded to the Polar Flow for Coach cloud platform via a mobile app on the participants’ phones. Additionally, participants will record their rating of perceived exertion in a provided logbook after each session using the modified Borg scale from 0 to 10 (0 indicates no effort, and 10 indicates maximal effort) [29].

### Overview of Study Procedures

Participants will complete a 6-week exercise intervention with 2 data collection visits to the laboratory at the pre- (week 0) and postintervention (week 7) time points. Before arrival on a data collection visit, participants will be asked to fast for 10 to 12 hours overnight.

The first data collection visit (week 0) will include a study briefing covering the purpose, procedures, roles, risks, and benefits of the study; informed consent and assent documentation; study questionnaires; dual-energy x-ray absorptiometry (DEXA) scans; a graded exercise test; a dried blood spot test; and an intervention briefing (6-week prescription and equipment instructions).

The second data collection visit (week 7) will include study questionnaires; a DEXA scan; a graded exercise test; a dried blood spot test; and a debriefing (reviewing pre- and posttest changes in outcomes).

### Home-Based Intervention

For the 6-week intervention, participants will be asked to achieve 4 days of XR exergaming per week for 60 minutes per day, totaling 240 minutes per week. This prescription aims to be consistent with a previous case study in which a child with spina bifida achieved a substantial reduction in weight from 187 minutes per week of exergaming at approximately 76% of the maximum heart rate [30]. A duration of 240 minutes per week was chosen for this study based on preliminary (nonpublished) findings from a recently completed trial [28]. A prescription of 240 minutes per week will likely result in 187 minutes per week due to time spent in warm-up, cooldown, and in-game level selection. Two participants will play the exergame standing up, and 2 participants will play seated in a chair or their own wheelchair. This will allow us to observe whether vigorous-intensity play can be achieved while sitting or standing. This methodology is an extension of our recently completed pilot trial [28], which demonstrated that moderate-intensity exercise can be achieved while XR exergaming from either a seated or standing position in young people with CP (GMFCS levels I-IV; findings not yet published).

**Figure 2.**
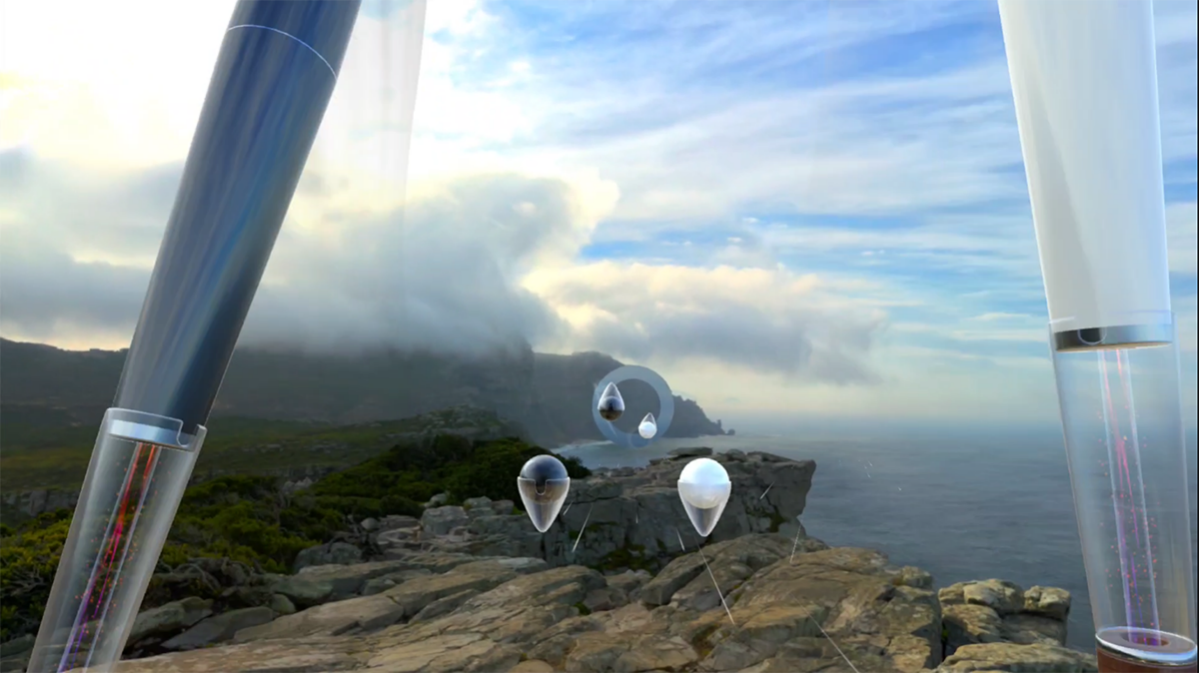
First-person perspective of Supernatural gameplay through the Meta Quest head-mounted display.

**Figure 3.**
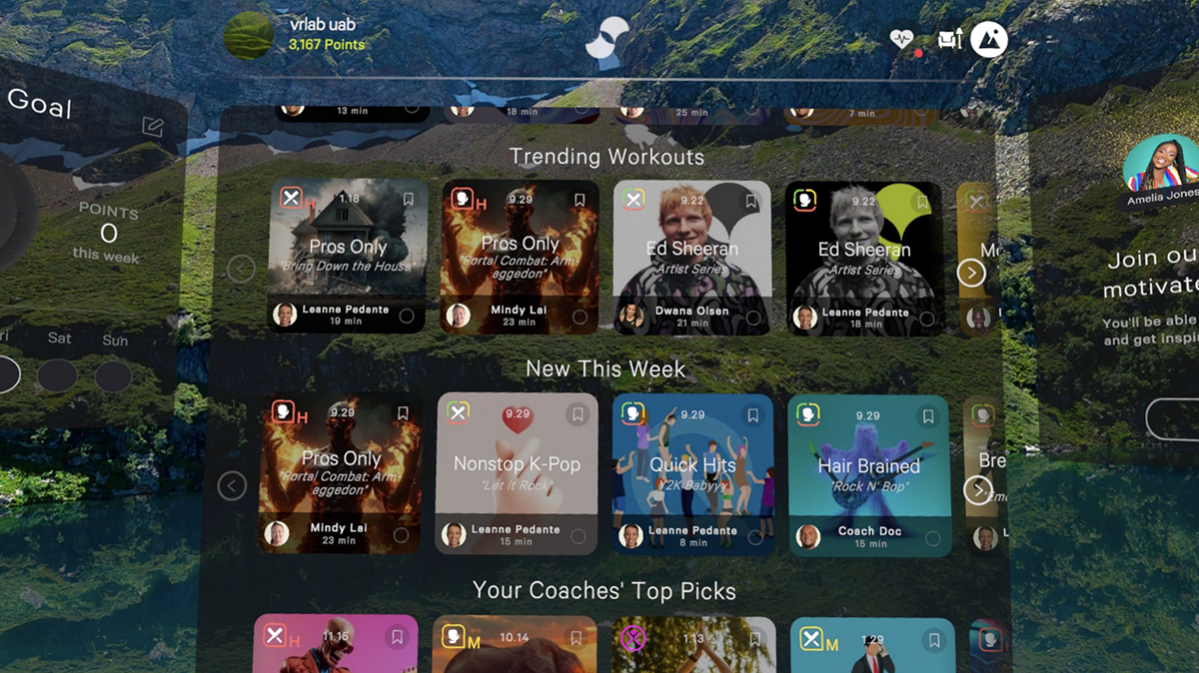
Example of exercise routines and music genres in the Supernatural game.

Participants will be asked to play the game Supernatural and perform high-intensity in-game routines to try to achieve a vigorous-intensity heart rate (>75% of the maximum heart rate [31] as determined by the Tanaka equation [32]). Supernatural is a live-service game designed to improve physical fitness through a variety of activities and programs (Figure 2). The initial setup includes accessibility options for seated gameplay and range of motion and vigorous-intensity programs and requires a monthly subscription to access its continually updated content. The game includes virtual, humanlike exercise trainers that provide verbal praise and instruction, as well as exercise routines that follow popular music from various genres (Figure 3).

To enhance safety, participants will be instructed on how to set up a play space using the Guardian feature of the headset, a virtual boundary that will halt play should the player exceed the space. Additionally, caregivers will be certified in Red Cross cardiopulmonary resuscitation and automated external defibrillator training by a certified instructor at baseline data collection and be provided with a home automated external defibrillator (Philips HeartStart OnSite Home automated external defibrillator) to use in the case of an emergency [33].

### Intervention Coaching

The intervention will incorporate low-dose behavioral physical education coaching via phone call. The purpose of the calls is to enhance adherence to the tele-exergaming program by providing a sense of accountability [26] while enhancing exercise literacy and mastery of the games to promote confidence [28]. Sessions will last 15 minutes and take place weekly. Participants will be accompanied by a caregiver as caregiver knowledge and attitudes significantly influence participation [34-36]. Specific strategies during phone calls will include introducing the importance of aerobic exercise for young people with disabilities; providing verbal praise for meeting exercise goals or smaller steps toward goals; reviewing the benefits of aerobic exercise based on systematic reviews; and solving technological, access, and logistical barriers to XR exercise. These strategies are underpinned by social cognitive theory [37]. Coaching will be delivered by research staff trained by the principal investigator (BL).

### Measures and Procedures

#### Questionnaires

The study will use 3 questionnaires. A questionnaire will be used to assess participant characteristics (eg, age, sex, and BMI). Diet will be recorded using the Adolescent Food Habits Checklist (week 0 and week 7) to account for changes in diet. This short survey includes 23 items in which the person can respond with “true” or “false” to a variety of healthy eating habit questions. The survey is scored using a frequency count of 1 point for each “healthy” response. The final score is calculated using the following equation: number of “healthy” responses × (23/number of items completed) [38]. The Godin Leisure-Time Exercise Questionnaire (GLTEQ) will be used to examine physical activity levels outside of the program (week 0 and week 7) to account for participation in extra physical activity that could affect study outcomes. The GLTEQ [39] is a 3-item self-report questionnaire that is used to measure habitual physical activity. The GLTEQ asks participants to report the number of times that low-, moderate-, and vigorous-intensity physical activity was performed for longer than 15 minutes in a typical week. The numbers reported for moderate and vigorous exercise intensity are then multiplied by 7 and 9, respectively, and summed to obtain a score, which is referred to as the health contribution score. A health contribution score of <24 is classified as physically inactive, whereas a score of ≥24 is considered physically active [40]. There is evidence to support the GLTEQ as a valid and reliable measure of physical activity among adults with multiple sclerosis [41] and adolescents [42].

#### Feasibility Outcomes (Aim 1)

Components of feasibility will be evaluated through quantitative metrics, namely, intervention safety, participant adherence, and technical issues. Adherence will be measured through minutes of exergaming performed per week throughout the study, which will be objectively recorded by the Polar Verity Sense monitor. Specific measures of exercise minutes will include two categories: (1) vigorous-intensity minutes per week divided by the desired target of 187 minutes and (2) total minutes per week. An arbitrary value of >70% attendance will be used as a cutoff for classification of “acceptable” adherence, a value of >80% will be used for classification as “good” adherence, and a value of >90% will be used to classify “excellent” adherence. Intervention safety will be measured through both adverse events and problem reports. To monitor safety, participants will be asked during coaching calls whether they experienced any minor problems (eg, joint pain or muscle soreness) or adverse events (eg, falls or injuries). Minor problems and adverse events (major problems) will be recorded and reported appropriately to the institutional review board of the University of Alabama at Birmingham. During weekly coaching calls, study staff will also record technical problems (frequency and type) that participants experience with either the XR or telehealth technology. Adverse events, problems, and technical issues will be descriptively reported, with no a priori criteria for classification as “feasible.”

#### Cardiometabolic Outcomes (Aim 2)

##### Aim 2 Primary Outcome

Body composition and weight will be measured using a DEXA scan. The primary outcomes from a whole-body DEXA scan will include body weight, total mass, and total fat mass in pounds, as well as total percentage of lean tissue and percentage of fat tissue. Participants will be instructed to lie on a DEXA scan table (Lunar iDXA; GE HealthCare) for a whole-body scan. Scans will be analyzed using the CoreScan software (GE HealthCare).

##### Aim 2 Secondary Outcomes

Indicators of cardiometabolic health will be measured via a dried blood spot test and sphygmomanometer. Specific outcomes will include resting blood pressure (systolic and diastolic), high-sensitivity C-reactive protein, hemoglobin A_1C_, fasting insulin, triglycerides, and cholesterol (total, low-density lipoprotein, and high-density lipoprotein). For the dried blood spot test, a lancet finger prick will be used to obtain 9 drops of blood on a paper card, which will be allowed to dry for several hours. Dried blood spot tests will be stored in an ultralow freezer and mailed in bulk for analysis to the ZRT Laboratory [43]. The ZRT Laboratory blood spot test has demonstrated excellent validity with venous serum samples [43,44].

### Cardiorespiratory Fitness Outcomes (Aim 2)

Cardiorespiratory fitness outcomes will include peak oxygen consumption measured using a portable metabolic cart (COSMED K5) during a graded exercise test on an arm ergometer, as well as maximal work output (measured in watts) and resting heart rate. The procedure for the graded exercise test will include a rest period, warm-up, graded exercise to exhaustion, and cooldown [33]. The rest period will last 2 to 3 minutes. Warm-up will include 1 to 2 minutes of cycling at a low, self-selected comfortable pace. For the graded exercise test, participants will be asked to maintain a speed of 60 revolutions per minute (or as close to 60 revolutions per minute as possible if 60 is not tolerable). Resistance will start at 0 watts and increase by 10 watts every minute until volitional fatigue or the participant meets 3 out of 5 of the following indirect criteria: a heart rate greater than 85% of the age-predicted maximum heart rate, self-reporting a score of 8 or higher on the Borg rating of perceived exertion scale from 0 to 10, demonstration of a respiratory exchange ratio of 1:1 or higher, a plateau in oxygen consumption, and subjective signs of exhaustion. Heart rate and oxygen consumption will be measured continuously. Participants will then have a slow, active cooldown until heart rate recovers.

### Intervention Acceptability Through Qualitative Inquiry (Aim 3)

Intervention “acceptability” will be evaluated qualitatively. Following completion of the intervention, we will conduct an in-person semistructured interview with the participants led by the study interventionist (BL). The qualitative component of the project will use an intrinsic case study design [45,46] to gain an in-depth understanding of the experiences of a single individual who completes the program. The qualitative component of the study will be underpinned by a critical realism ontological perspective and an interpretivism epistemological perspective [47,48]. These philosophical assumptions will allow the research team to acknowledge that the participant will describe their perceived reality while also recognizing that these accounts may be shaped by interviewer-participant interaction and subsequent interpretive analysis. Consistent with interpretivist traditions, the intention of this component will be to explore how the participant makes sense of engaging in extended reality rather than to quantify code frequencies or identify a singular objective truth.

The interview guide will contain approximately 10 core questions with relevant probes. These prompts will elicit perspectives on overall impressions of the program, perceived strengths and limitations, experiences using VR for upper-extremity exercise, comparison with previous rehabilitation experiences, barriers to and facilitators of meeting the intervention prescription, preferences, and suggestions for refining the protocol in preparation for a larger trial. The interviews will be conducted by a qualitative expert in disability and exercise (BL) who has extensive experience conducting interviews in this area (>500 interviews). All interviews will be audio recorded and transcribed verbatim by a member of the research team.

### Data Analyses

Descriptive statistics will be obtained for all study variables, including means, SDs, effect sizes, graphs, and 95% CIs. Quantitative outcomes across time will be graphically reported. Qualitative analysis will include thematic analysis.

Qualitative data analysis will involve 2 analysts and follow the 6-step approach by Braun and Clarke [49,50] to latent thematic analysis. First, the analysts will familiarize themselves with the transcripts through repeated readings and early reflexive noting of tone, context, and emerging ideas. Second, initial codes will be generated across the transcript to capture meaningful units related to actions, perceptions, and emotions. Third, codes will be organized into a single analytic level of themes given the small study sample size and the aim to privilege depth over breadth [51]. Fourth, candidate themes will be reviewed against the full dataset to ensure coherence and alignment with the study aims. Fifth, themes will be refined to clarify their central concepts, and illustrative quotes will be identified based on conceptual richness rather than frequency. Sixth, findings will be synthesized for an interpretive report and supported by participant quotes. Throughout the analytic process, rigor will be strengthened through reflexive memoing and maintenance of an audit trail documenting coding and theme development. The transcripts will be checked for accuracy by a second member of the research team to ensure fidelity before analysis.

### Ethical Considerations

The protocol and informed consent and assent forms were approved by the Institutional Review Board for Human Use of the University of Alabama at Birmingham (IRB-300014468) on September 3, 2025. Prospective participants will provide written informed consent or assent documentation before taking part in the study. Consent and assent forms will be completed on the baseline data collection visit. Participants will receive an electronic gift card that will be loaded with US $590 for completing the data collection visits. The rationale for this value is to account for the commitment and time required of both the child and caregiver to complete the study. This trial has been registered on ClinicalTrials.gov (NCT07155213).

## Results

This study was approved by the university institutional review board on September 3, 2025. The study was initiated in October 2025, and the first participant was enrolled in October 2025. All data are anticipated to be collected by December 2025. The study end date is April 4, 2026.

## Discussion

### Expected Findings

This study will explore the potential impact of high-intensity arm exercise training using XR on both cardiorespiratory fitness and cardiometabolic health in 4 young people with CP. An effective arm exercise program could be extremely beneficial for this population because evidence to support that exercise can reduce body weight or other indicators of cardiometabolic health among young people with CP is limited [11,23,52]. People who have difficulty walking have few options for health-enhancing aerobic exercise [23,24,52,53].

### Strengths and Limitations

Considering that this program uses telehealth communication strategies and “off-the-shelf” technology that can be purchased at any major electronics retailer, it has the potential to be carried forward in a large randomized controlled trial for young people with CP. This strength could assist in reaching larger sample sizes that could lead to more confirmatory findings from exercise trials. The average sample size of children and youth with CP for a randomized controlled trial is 27, and one of the largest trials included 159 people [52], sample sizes that are not generalizable to the diverse needs of people with CP [54]. Another strength of this study is that it includes age-appropriate games, themes, and music, potentially leading to high levels of prolonged engagement [55]. This is a crucial benefit given that improvements to cardiometabolic health require higher volumes of exercise over long periods [16,17]. Moreover, the XR exergaming can be done asynchronously at home without supervision of an exercise specialist, which eliminates the need for transportation to an on-site facility and reduces burden on research staff.

A notable limitation of this study is the small sample size. A second major limitation of this study is the requirement for on-site data collection procedures, which will hinder the scale-up potential of the program in a future study. On-site visitations are inconvenient for young people with CP and their caregivers due to a lack of nearby testing facilities and lack of time with busy school schedules and daily activities [10,56]. Thus, there is a need to develop remote data collection procedures with strong psychometric properties that can complement the remote nature of an XR telehealth program. The blood spot test can be performed remotely by shipping blood spot test kits to the home. However, to the best of our knowledge, there is no sound home-based method for measuring cardiorespiratory fitness or body composition imaging among people with mobility disabilities [20].

### Conclusions

This case study examines whether intensive XR exergaming produces short-term improvements in key health indicators among a small cohort of young people with CP. If the aims of this study are supported, a larger pilot trial will be warranted.

## Supplementary material

10.2196/85246 Peer Review Report 1 Peer review report by the Center for Engagement in Disability Health and Rehabilitation Sciences (CEDHARS) at the University of Alabama at Birmingham.

## Abbreviations

CP: cerebral palsy
DEXA: dual-energy x-ray absorptiometry
GLTEQ: Godin Leisure-Time Exercise Questionnaire
GMFCS: Gross Motor Function Classification System
HMD: head-mounted display
XR: extended reality
