# Supplementary material for: Feasibility of Vigorous Extended Reality Tele-Exergaming for Cardiometabolic Health in Youth With Mobility Disabilities: Protocol for a Case Series Study
Source: JMIR Res Protoc. 2026 Feb 27;15:e85246. doi: 10.2196/85246 (PMC12954695; doi:10.2196/85246)
Supplement: Peer Review Report 1 [file resprot-v15-e85246-s001.pdf]

Reviewer questions

Byron Lai "*Feasibility of a Vigorous Extended Reality Exergaming for Cardiometabolic Health in Children with Mobility Disabilities: A Case Series*"

These questions must be addressed and submitted in writing prior to *the first meeting with the CEDHARS staff to receive funding. Please submit your response via email to* [redacted].

1. Please address the concern raised by reviewer 1 regarding the lack of a plan to address adherence and compliance verification.
2. Please respond to the comment from Reviewer 3 regarding the role of dietary intake "*It is unclear whether exercise alone for 6 weeks will be enough to stimulate weight loss, especially without a diet component. As an example, the individual could eat more because they are working out at a higher intensity, confounding the results*."
3. Please clarify the inconsistence mentioned by several reviewers regarding the length of intervention, 4 or 6 weeks of training.

CEDHARS Pilot Grant Funding
Reviewer Score Sheet

Application Title: Feasibility of a Vigorous Extended Reality Exergaming for Cardiometabolic Health in Children with Mobility Disabilities: A Case Series
PI: Byron Lai

The review process for the CEDHARS pilot grant applications followed the **Simplified NIH Peer Review Framework.** Each proposal was reviewed by 5 UAB faculty members, who were asked to provide scores based on the following criteria:

Scored Review Criteria

1. **Overall Impact Score**: Reflects reviewer's assessment of the likelihood for the project to exert a sustained, powerful influence on the research field involved, in consideration of Factors 1-3 scores.
2. **Factor 1 Score**: Importance of the Research (Scored 1-9). Evaluates significance and innovation. Addresses how important the proposed research is in advancing scientific knowledge an impact.
3. **Factor 2 Score**: Rigor and Feasibility (Scored 1-9). Evaluates approach. Assesses how rigorous and feasible the proposed methods are in addressing the research question.
4. **Factor 3 Score**: Expertise and Resources (Evaluated as Sufficient/Not Sufficient). Evalutes investigator and environment. Determines whether the applicant's expertise and resources are adequate to complete the proposed research. If deemed insufficient, reviewers must provide an explanation.

Your **average overall impact score was 3.20**. Based on the NIH scoring system, scores between 1–3 reflect exceptionally strong applications with minimal weaknesses, scores between 4–6 indicate good applications with moderate concerns, and scores between 7–9 suggest significant weaknesses that limit the project's potential impact.

We have provided each reviewer's feedback below to support your continued development. You are not required to address these individual comments for funding purposes; only the specific questions outlined in your award letter must be addressed. These comments are shared solely for your learning and future improvement.

If you have any questions or concerns about the reviewer feedback, please do not hesitate to reach out to [redacted]

Reviewer 1

**Overall Impact Score**: 3
See comments in the below sections.

**Factor 1 Score**: 3
Strengths:<br>Aerobic exercise solutions for youth with mobility disabilities is needed.<br>Use of XR exergaming as a way to address this need among youth with disabilities.<br><br>Weaknesses:<br>Lack of blood biomarker changes and unable to replicate body weight reduction of an earlier success.<br>Most likely any success would be temporal.

**Factor 2 Score:** 4
Strengths<br>This work builds off an NIH R03 application, good preliminary work.<br>Adequate safety precautions and caregiver training<br>Use of appropriate biomarker outcomes<br><br>Weaknesses<br>For Study 1, only 4 participants (2 per group) and 1 person can greatly skew data for better or worse<br>DEXA as primary outcome measure but previous work showed no change in weight, doubtful that 6 wks of 1hr of exercise 4x a week will result in DEXA changes at the level of energy expenditure spent, as blood biomarkers did not change either.<br>Unable to replicate meaningful body weight reductions.<br>Hypothesis 1 mentions 4 wks of training elsewhere it is 6 wks<br>Adherence will likely be an issue, and did not see a plan to address<br>Participants will likely become bored with repetitive playing of a game over 6 weeks for 1 hr, 4 days/wk.<br>Lack of compliance verification

**Factor 3 Score**: Satisfactory
N/A

Reviewer 2

**Overall Impact Score**: 2
Strong proposal that is very well aligned with the CEDHARS Pilot Grant program and draws upon the experience and prior work of the investigative team. A few minor concerns regarding study

procedures that speak to the rigor of the work but these are likely issues of omission more so than problems per se.

Factor 1 Score: 2

Strengths:
- Clearly aligned with the CEDHARS Pilot Grant program with its focus on youth with mobility disabilities.
- Builds on previous research done by the PI by addressing a gap (i.e., identifying the right dose of exercise intervention) that has emerged as a result of that work. Very innovative in its application of extended reality to address a clear and substantial barrier for this population.

Weaknesses:
- It was a little unclear whether/how Aim 2 differed from Aim 1, other than the outcome being used, and why that might be important.
- The proposal mentions a "need to identify community strategies that promote early exercise adoption". I wasn't sure what was meant by "community strategies". Is that what this is? In what ways is the community involved in this research?

Factor 2 Score: 2

Strengths:
- Investigator has experience with the proposed approach, including the equipment used in the intervention.
- Clear description of intervention.

Weaknesses:
- Some details are not described in the proposal (e.g., qualitative analysis) although are referenced as those used in previous work.
- It was a little hard to discern the intervention/exercise period. E.g., aim 1 mentions 6 weeks of vigorous-intensity XR exergaming, while the hypothesis mentions improvement in cardiometabolic health after 1 month of vigorous intensity XR exergaming.

Factor 3 Score: Satisfactory

N/A

<u>Reviewer 3</u>

Overall Impact Score: 3

-There is a greater recognition that high intensity exercise can lead to greater health related outcomes, which is a strength of this study
-The small number of participants is a concern for reproducibility however for this type of grant, is it unrealistic to have more participants for a multi-week intervention.

**Factor 1 Score**: 3
-Managing body mass and composition is a challenge for individuals with disabilities who may not have feasible options to engage in regular physical exercise creating a significant need that the current study addresses.<br>-Creating safe interventions that can be performed at home in a gamified way where intensity can be scaled is important.<br>-It is unclear whether exercise alone for 6 weeks will be enough to stimulate weight loss, especially without a diet component. As an example, the individual could eat more because they are working out at a higher intensity, confounding the results.

**Factor 2 Score**: 3
-Behavioral coaching will accompany the intervention, which is a strength<br>-While this is the state of the art with home interventions, it is unclear how rigorous these interventions are such that they could be repeated with fidelity. Instead, it seems that everything is dependent on the person and how they are able to interact with the game.<br>-Outcomes are appropriate for the intervention utilized.<br>- The small number of participants is a concern for reproducibility

**Factor 3 Score**: Satisfactory
N/A

<u>Reviewer 4</u>

**Overall Impact Score**: 7
See below sections for strengths and weaknesses of each factor. There is an inconsistent use of "XR" and "ER" for extended reality in the Aims page. There is also a lot of fluctuating between "YwMD" and "YwCP" which can be hard to decipher – "CwCP" is also used without explanation. There are three figures in the application, labelled as Figure 2, 5 and 11. However, the text refers only to Figure 4. This is poor grantsmanship.

**Factor 1 Score**: 8
Strengths: Increasing cardiovascular health in youth with mobility issues can be difficult and is a high priority for general health and quality of life. Use of exergaming to engage young people is an advantage.<br><br>Weaknesses: Seem to be limited novelty or innovation based on the current studies being done by the PI and colleagues. It seems that almost the identical study is being done as an R03 with less intense exercise. Thus, an incremental advancement, given that the results of the R03 are under review.

**Factor 2 Score**: 7

Strengths: A similar protocol is already in place for a current project, suggesting that it is feasible.<br><br>Weaknesses: Having high-responding individuals as potential participants seems like it will sway the results. The small number of participants (n=4) makes it highly unlikely to result in statistically meaningful outcomes. There are no plans for missing data, which can have a major impact on a sample of this size.

**Factor 3 Score**: Satisfactory
N/A

Reviewer 5

**Overall Impact Score**: 1
The application is based on continued work of the PI and team, it addresses an important topic, and for a pilot the approach is appropriate.

**Factor 1 Score**: 1
The project expands novel use of extended reality technology for examining acceptability of vigorous intensity protocol for youth with mobility disabilities

**Factor 2 Score**: 1
More so than in moderate activity, in a vigorous intensity intervention, adherence to the protocol is crucial, and the proposal includes a behavioral coaching component to address adherence

**Factor 3 Score**: Satisfactory
N/A
